# Dietary Diversity Score and Its Sociodemographic Determinants in School-Aged Children: Cross-Sectional Baseline Findings From a Quasi-experimental Study

**DOI:** 10.7759/cureus.85804

**Published:** 2025-06-11

**Authors:** Tulika Singh, Sanjay Kumar, Setu Sinha, Shishir Kumar

**Affiliations:** 1 Community Medicine, Indira Gandhi Institute of Medical Sciences, Patna, IND

**Keywords:** dietary diversity, micronutrients, nutrition, public health, quasi-experimental study, school-age children

## Abstract

Background

Dietary diversity is an essential component of nutritional adequacy, particularly for school-aged children who are undergoing rapid physical and cognitive development. Inadequate dietary diversity can lead to micronutrient deficiencies, undernutrition, and poor health outcomes. Understanding the factors influencing dietary diversity among children is essential for designing effective interventions. This study aims to assess dietary diversity and its sociodemographic correlates among school-aged children as part of a larger quasi-experimental trial.

Methods

This cross-sectional study was conducted among 202 school-aged children (9-12 years old) in a socioeconomically disadvantaged urban setting. Data were collected as part of a larger quasi-experimental study designed to evaluate a school-based nutritional intervention. Only baseline data were analyzed for this paper. A structured questionnaire was administered to capture sociodemographic details, nutritional knowledge, attitudes, and dietary intake through a 24-hour recall. Dietary diversity scores (DDS) were calculated based on the Food and Agriculture Organization (FAO) guidelines. Anthropometric data and sociodemographic variables were also recorded. Descriptive and inferential statistics were used to assess associations.

Results

The mean DDS was 4.35 ± 1.07. Approximately 46.5% of students had low dietary diversity (DDS < 5). Most children consumed starchy staples (95.5%) and dairy products (85.6%), whereas consumption of organ meats and green leafy vegetables was low. There were no statistically significant associations between DDS and gender (p = 0.216), caregiver education (p = 0.510), or socioeconomic status (p = 0.404).

Conclusion

The overall dietary diversity among school-aged children was suboptimal, with limited intake of several nutrient-rich food groups. The lack of association with sociodemographic variables suggests broader environmental or cultural influences on dietary patterns. These findings underscore the need for school-based nutrition education and policy measures to promote diverse, balanced diets among children in disadvantaged regions.

## Introduction

Ensuring optimal nutrition during childhood is essential for supporting physical growth, cognitive development, immune competence, and overall well-being. School-aged children (6-12 years) experience rapid developmental changes, making this period critical for nutritional interventions. Among various indicators of diet quality, dietary diversity, the variety of foods consumed across multiple food groups within a reference period, has emerged as a validated proxy for micronutrient adequacy and general diet quality. A more diversified diet typically ensures sufficient intake of essential nutrients, thereby reducing the risk of undernutrition, stunting, and micronutrient deficiencies [1,2].

In India, especially in economically disadvantaged settings, children's diets are frequently monotonous, with an overreliance on starchy staples and limited intake of fruits, vegetables, and animal-source foods. The National Family Health Survey (NFHS-5) highlighted persistent dietary inadequacies and poor nutritional status among school-aged children, which continue to challenge national and state-level efforts to combat malnutrition [3]. These dietary patterns contribute to both macro- and micronutrient deficiencies, which can impair growth, cognitive functioning, and resistance to infections [2,4,5].

Bihar is one of the most socioeconomically disadvantaged states in India, characterized by high poverty rates, low literacy, inadequate healthcare infrastructure, and significant health disparities. NFHS-5 data report that Bihar has one of the highest burdens of stunting (42.9%), underweight (41%), and wasting (22.9%) among children under five, indicators reflective of poor nutritional environments [3]. The situation among school-aged children is likely to be equally concerning. Limited access to quality nutrition, especially in public school settings dependent on midday meals and familial resources, exacerbates these issues. These disparities necessitate context-specific interventions informed by empirical data from regions such as Bihar.

Studies conducted globally support the importance of dietary diversity in child nutrition. Arimond and Ruel found dietary diversity to be strongly associated with micronutrient adequacy in children across 11 countries [4]. Amugsi et al. demonstrated that maternal characteristics and household factors were linked to better child growth through dietary diversity in sub-Saharan Africa [6]. Flores-Vázquez et al. systematically reviewed educational nutrition interventions based on behavioral theories and found positive effects on adolescent dietary behaviors [7]. Jones et al. highlighted the association between WHO dietary diversity indicators and child growth outcomes [8]. In India, Ghosh et al. stressed the importance of dietary quality in preventing undernutrition [9]. De Onis and Branca emphasized the global burden of stunting linked to poor diet diversity [10], while Ruel et al. advocated for nutrition-sensitive interventions to improve child health outcomes [11]. These findings collectively underline the importance of improving dietary diversity to combat malnutrition.

Despite this growing evidence base, research on dietary diversity and its determinants among school-aged children in India remains limited, especially in states with high rates of malnutrition. This study seeks to address this gap by examining dietary diversity patterns among children aged 9-12 years. Although the data presented are derived from the baseline of a larger quasi-experimental trial, the present analysis focuses exclusively on the cross-sectional findings. By examining the associations between dietary diversity and key sociodemographic variables, the study aims to inform targeted nutrition interventions and policy initiatives. The objective of this study was to evaluate dietary diversity and examine its associations with key sociodemographic factors among school-aged children in a disadvantaged urban setting, using baseline data from a larger quasi-experimental study.

## Materials and methods

Study design and setting

This study is a cross-sectional analysis based on baseline data from a quasi-experimental study aimed at evaluating the impact of a school-based nutritional health education intervention. The study was conducted in public schools located in a socioeconomically disadvantaged urban setting. Schools were purposively selected after receiving written approval from the school authorities.

Participants and sample size

A total of 202 children aged 9 to 12 years, enrolled in grades 4 to 6 from selected schools, were included in the baseline survey. The inclusion criteria included children present during data collection and whose parents provided informed consent. Children with known medical or developmental conditions that might influence dietary patterns, those absent during the data collection period, and those for whom parental consent was not available were excluded from the study.

Data collection and tools

Data were collected using a structured, pretested questionnaire administered by trained field investigators. The questionnaire gathered the following information.

Sociodemographic Characteristics

Age, sex, religion, caregiver education, occupation, and socioeconomic status (SES) were assessed using the BG Prasad Scale.

Dietary Intake

Dietary data using a 24-hour recall, capturing consumption from the nine Food and Agriculture Organization (FAO)-defined food groups. The dietary diversity score (DDS) was calculated by summing the number of food groups consumed in the preceding 24 hours. Based on the FAO guidelines, a DDS of <5 was categorized as low dietary diversity, while a score of ≥5 was considered adequate [12].

Anthropometric Measurements

The anthropometric measurements, including height, weight, and mid-upper arm circumference (MUAC), were conducted in a designated area within the school premises, often in an available room or classroom, depending on the school's infrastructure. Height was measured using a portable stadiometer (to the nearest 0.1 cm), and weight was recorded using a calibrated digital weighing scale (to the nearest 0.1 kg). MUAC was measured using a nonstretchable measuring tape. All measurements were taken by trained staff following standardized procedures to ensure accuracy and reliability across schools.

Statistical analysis

Data were entered into MS Excel (Microsoft Corporation, Redmond, Washington, United States) and analyzed using IBM SPSS Statistics for Windows, Version 16 (Released 2007; IBM Corp., Armonk, New York, United States). Descriptive statistics (mean, SD, frequencies) were used for summary variables. Independent sample t-tests and chi-square tests were applied to assess associations between DDS and independent variables. A p-value of <0.05 was considered statistically significant.

Ethical considerations

Ethical approval was obtained from the Institutional Ethics Committee. Written informed consent was obtained from the caregivers, and assent from the children. Data confidentiality and participant anonymity were strictly maintained.

## Results

Out of the 202 children, 65.3% were male and 34.7% were female, with a mean age of 10.42 ± 1.14 years. The majority (90.8%) belonged to the Hindu religion. Primary caregivers were predominantly mothers (80.7%). Most caregivers had education up to the secondary level (47.0%) and were homemakers (24.7%) or engaged in the private sector (30.7%). About 55.4% of the families belonged to the middle-class socioeconomic status (Table 1).

**Table 1:** Sociodemographic characteristics of the study participants (N = 202), socioeconomic status (SES) as per BG Prasad Scale

Variable	Category	Frequency (n)	Percentage (%)
Age (years)	Mean ± SD	10.4 ± 1.1	
Sex	Male	132	65.3
	Female	70	34.7
Class	IV	72	35.6
	V	68	33.7
	VI	62	30.7
Primary caregiver	Mother	163	80.7
	Father	14	6.9
	Other guardian	25	12.4
Caregiver education	Primary	74	36.6
	Secondary	95	47.0
	Higher secondary	26	12.9
	Graduate and above	7	3.5
Caregiver occupation	Homemaker	50	24.7
	Government job	44	21.8
	Private sector	62	30.7
	Self-employed	46	22.8
Socioeconomic status	Upper	14	6.9
	Upper middle	46	22.8
	Middle	112	55.4
	Lower middle	30	14.9

The mean DDS among participants was 4.35 ± 1.07. Approximately 46.5% of participants had a DDS of less than 5, indicating inadequate dietary diversity. The most commonly consumed food groups were starchy staples (95.5%) and legumes, nuts, and seeds (74.3%). Organ meats (5.9%) and dark green leafy vegetables (31.7%) were the least consumed (Table 2).

**Table 2:** Mean dietary diversity score (DDS) and consumption by food group* *multiple response table

Food group	Consumed (n)	Percentage (%)
Starchy staples	193	95.5
Dark green leafy vegetables	64	31.7
Vitamin A-rich fruits and vegetables	91	45.1
Other fruits and vegetables	106	52.5
Organ meat	12	5.9
Meat and fish	67	33.2
Eggs	53	26.2
Legumes, nuts, and seeds	150	74.3
Milk and milk products	173	85.6
Mean DDS ± SD	4.35 ± 1.07	

**Figure 1:**
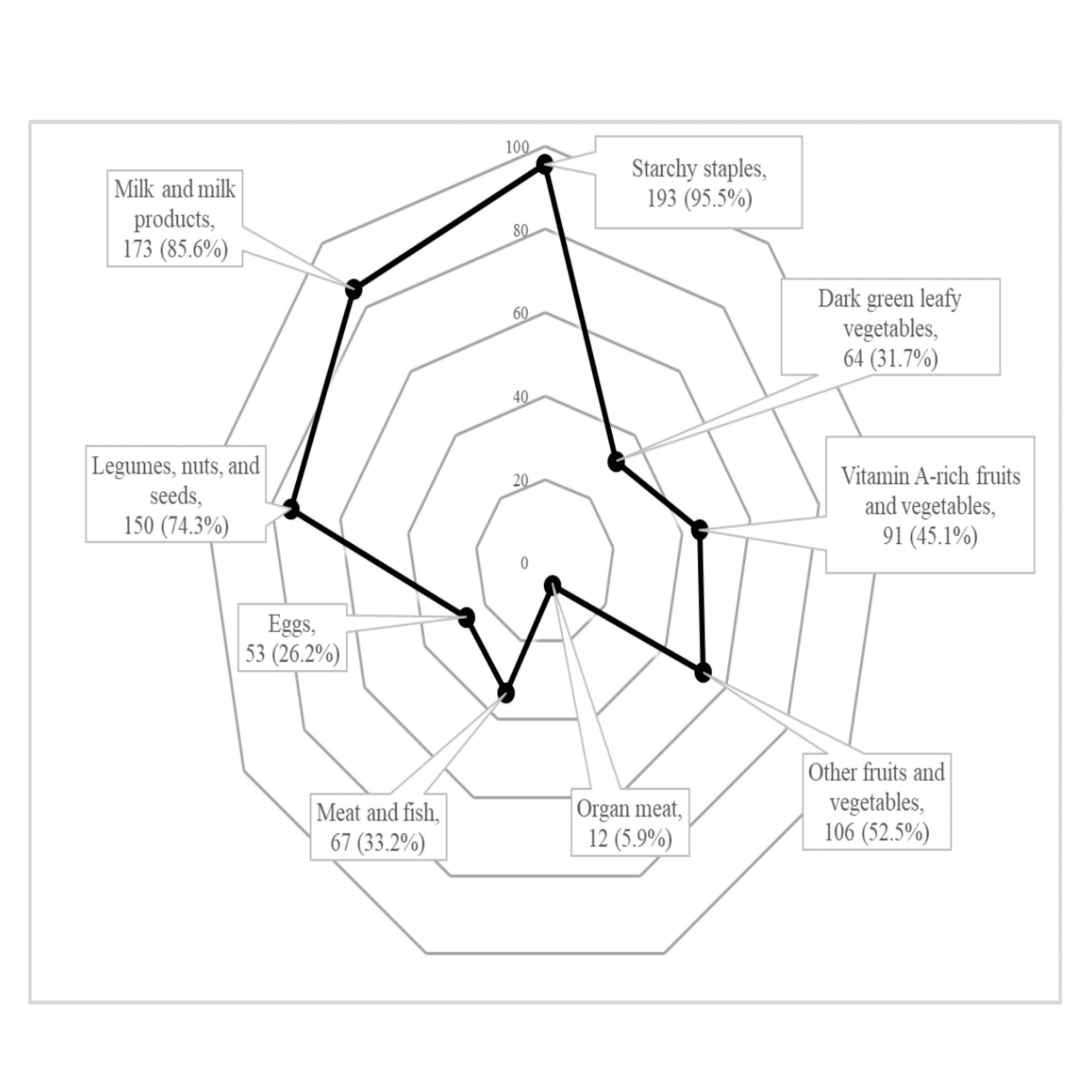
Dietary diversity profile by food groups among study participants (n = 202) Multiple response dataset

DDS was not significantly associated with gender (p = 0.216), caregiver education (p = 0.510), or socioeconomic status (p = 0.404) (Table 3). In Figure 1, the radar chart illustrates the percentage of children who consumed each of the nine food groups used to calculate dietary diversity scores, as per the FAO guidelines.

**Table 3:** Association between sociodemographic factors and dietary diversity score of the children p < 0.05 = significant

Variable	Category	Mean DDS ± SD	p-value
Sex of the child	Male	4.44 ± 1.12	0.140
	Female	4.19 ± 0.96	
Caregiver education	Up to primary	4.26 ± 1.12	0.426
	Secondary and above	4.40 ± 1.04	
Socioeconomic status	Middle and below	4.21 ± 1.12	0.246
	Upper middle and above	4.41 ± 1.05	

The mean weight was 35.9 ± 7.27 kg, the mean height was 142.1 ± 5.12 cm, the BMI was 17.8 ± 3.04, and the MUAC was 20.1 ± 2.31 cm.

## Discussion

This study presents a cross-sectional assessment of dietary diversity among school-aged children in a socioeconomically disadvantaged setting. The mean DDS of 4.35, with nearly half the participants falling below the threshold for adequate dietary diversity, underscores a significant concern in dietary quality. It reflects poor diet quality and potentially inadequate micronutrient intake.

The findings are consistent with previous literature indicating suboptimal dietary patterns among children in low-resource settings [2,3]. Arimond and Ruel’s multicountry analysis showed strong associations between dietary diversity and improved micronutrient adequacy [4]. These results are comparable to findings by Amugsi et al. and Gatica-Domínguez et al., where limited dietary diversity correlated with poor nutritional outcomes in children across low- and middle-income countries (LMICs) [6,13].

In the present study, DDS was not significantly associated with caregiver education or SES, indicating that other contextual or structural barriers might influence dietary behaviors. This aligns with findings by Ghosh et al., who argued that dietary quality in India is affected by factors beyond economic access, including food culture, availability, and awareness [9].

The limited consumption of nutrient-dense foods, particularly organ meats, green leafy vegetables, and vitamin A-rich fruits and vegetables, raises public health concerns. These groups are critical sources of essential vitamins and minerals. According to the FAO guidelines, inadequate intake from these categories can contribute to deficiencies in iron, vitamin A, and other micronutrients [1]. The reliance on carbohydrate-rich staples suggests a need for policy interventions to improve food access and awareness of balanced diets. The Global Nutrition Report, similarly, emphasizes that poor diet quality is now a greater health risk than undernutrition alone [2].

Interventions aimed at improving nutrition literacy among children and caregivers may be beneficial. Flores-Vázquez et al. found that school-based, theory-driven educational programs led to significant improvements in adolescent dietary choices [7]. Ruel et al. advocate integrating nutrition-sensitive approaches across health, agriculture, and education sectors [11]. Such strategies, including community kitchens, diversified school meals, and food fortification, may help mitigate dietary deficiencies in contexts like ours.

Although this study was embedded within a larger quasi-experimental design, its current findings offer a valuable baseline understanding of dietary diversity in a high-risk population. However, certain limitations must be acknowledged, including reliance on self-reported dietary recall, potential recall bias, and purposive sampling.

## Conclusions

This cross-sectional analysis, forming the baseline of a larger quasi-experimental study, found that nearly half of the surveyed school-aged children had inadequate dietary diversity, with minimal consumption of key micronutrient-rich food groups. Starchy staples dominate the daily intake, while consumption of nutrient-dense food groups remains inadequate. No significant associations were observed between DDS and sociodemographic factors. These findings underscore the need for multisectoral, context-specific, evidence-based nutrition interventions at the school and community levels to improve dietary quality among children. Future longitudinal studies should assess the impact of targeted interventions on diet and health outcomes in similar settings.

## Appendices

Dietary Diversity Score and Its Sociodemographic Determinants in School-Aged Children: Cross-Sectional Baseline Findings From a Quasi-Experimental Study

Questionnaire

 Questionnaire No.________

Section 1: Demographic information

1.   Full Name:

2.   Age (Years):                                                           3. Gender:

4. Class:                                                                     5. Section:

6. Religion:      Hinduism                   Islam                    Sikhism                      Christianity

7. Do you have any siblings?     Yes                    No

  If yes, how many?  1              2                     3                  4                >4

Section 2: Knowledge about healthy eating 

Section 3: Attitude toward healthy eating

Section 4: Diet diversity questionniare

**Table 4:** Dietary diversity (baseline) (1) FAO/Nutrition and Consumer Protection Division, version of February 2007. Please acknowledge FAO in any documents pertaining to use of this questionnaire (2) This questionnaire may be used for any individual above the age of three years. For children under three, the dietary diversity questionnaire used in DHS surveys for young children is more appropriate

Individual dietary diversity questionnaire 1, 2			
Please describe the foods (meals and snacks) that you ate yesterday during the day and night, whether at home or outside the home. Start with the first food eaten in the morning			
Q. No.	Food group	Examples	Yes = 1, No = 0
1	Cereals	Rice, chapati, roti, parantha, puri, bread, or any other foods made from millet, sorghum, maize, rice, wheat, or other locally available grains	
2	Vitamin A-rich vegetables and tubers	Pumpkin, carrots, squash, or sweet potatoes that are yellow or orange inside + other locally available vitamin A-rich vegetables	
3	White tubers and roots	White potatoes, white yams, or foods made from roots	
4	Dark green leafy vegetables	Palak, methi,bathua, dark green/leafy vegetables, including wild ones + locally available vitamin A-rich leaves	
5	Other vegetables	Other vegetables, including wild vegetables	
6	Vitamin A-rich fruits	Ripe mangoes, papayas + other locally available vitamin A-rich fruits	
7	Other fruits	Other fruits, including wild fruits	
8	Organ meat (iron-rich)	Liver, kidney, heart, or other organ meats or blood-based foods	
9	Flesh meats	Chicken, duck, lamb, goat, or other birds	
10	Eggs		
11	Fish	Fresh or dried fish or shellfish	
12	Legumes, nuts, and seeds	Beans, peas, lentils, nuts, seeds, or foods made from these	
13	Milk and milk products	Milk, cheese, yogurt, or other milk products	
14	Oils and fats	oil, fats, or butter added to food or used for cooking	
15	Sweets	Sugar, honey, sweetened soda, or sugary foods such as chocolates, sweets, or candies	
16	Coffee/tea	Tea (black, green, herbal) or coffee	
			Yes = 1, No = 0
B.	Did you eat anything (meal or snack) outside the home yesterday?		
